# Towards a 384-channel magnetoencephalography system based on optically pumped magnetometers

**DOI:** 10.1162/IMAG.a.1042

**Published:** 2025-12-23

**Authors:** Holly Schofield, Ryan M. Hill, Lukas Rier, Ewan Kennett, Gonzalo Reina Rivero, Joseph Gibson, Ashley Tyler, Zoe Tanner, Frank Worcester, Tyler Hayward, James Osborne, Cody Doyle, Vishal Shah, Elena Boto, Niall Holmes, Matthew J. Brookes

**Affiliations:** Sir Peter Mansfield Imaging Centre, School of Physics and Astronomy, University of Nottingham, University Park, Nottingham, United Kingdom; Cerca Magnetics Limited, Nottingham, United Kingdom; QuSpin Inc., Louisville, CO, United States

**Keywords:** magnetoencephalography (MEG), optically pumped magnetometer (OPM), imaging hardware

## Abstract

Magnetoencephalography using optically pumped magnetometers (OPM-MEG) is gaining traction as a neuroimaging tool, with potential for improved performance and practicality compared with conventional instrumentation. However, OPM-MEG has so far lagged conventional-MEG in terms of the number of independent measurements of the MEG signal that can be made across the scalp (i.e., the number of channels). This is important since increasing the channel count offers improvements in sensitivity, spatial resolution, and coverage. Unfortunately, increasing channel count also poses significant technical and practical challenges. Here, we describe a new OPM-MEG system which exploits 3-axis sensors and integrated miniaturised electronic control units to measure MEG data from up to 384 channels. We also introduce a high-speed calibration method to ensure that the fields measured are high fidelity. We initially validate this system using a phantom: specifically, we localise a set of magnetic dipoles and compare the MEG-derived distances between them with the ground truth, demonstrating an accuracy of ~1 mm. We further show that the correlation between a mathematical model of the phantom and the magnetic fields measured is >0.998. Secondly, we demonstrate utility of our system for human MEG acquisition. Via measurement of visual cortex activity, we demonstrate improvements in sensitivity afforded by a higher channel count, and via a movie-watching experiment we show increased spatial resolution. In sum, we report the first OPM-MEG system with a channel count larger than that of typical conventional MEG devices. This represents a significant step towards OPMs becoming the sensor of choice for MEG.

## 1 Introduction

Optically pumped magnetometers (OPMs) are a newly emerging class of magnetic field sensor in the field of biomagnetism (see Brookes et al., 2022; Schofield et al., 2023 for reviews). OPMs are small, lightweight, easy to use, and can measure magnetic fields with high sensitivity. Consequently, they are ideal for measuring the small magnetic fields generated by the human body. For magnetoencephalography (MEG) (measuring magnetic fields generated by the brain), OPMs have significant advantages over the more widely used superconducting quantum interference devices (SQUIDs). Specifically, (1) non-cryogenic sensors get closer to the scalp, measuring fields that are larger in amplitude and less spatially diffuse (Boto et al., 2016; Iivanainen et al., 2017); (2) lightweight sensors can be mounted in a helmet which moves with the head, enabling participant movement during a scan (Boto et al., 2018); (3) unlike cryogenic sensors which are fixed in a “one-size-fits-all” helmet, OPMs can be mounted in helmets that accommodate multiple head sizes (babies to adults) (Hill et al., 2019). These advantages have been exploited in the study of babies (Corvilain et al., 2024) and neurodevelopment (Rhodes et al., 2025; Rier et al., 2024); to explore brain function underlying natural movement (O’Neill et al., 2025; Spedden et al., 2025); and to improve sensitivity (e.g., to detect epileptic spikes; Feys et al., 2022). Commercial OPM-MEG systems can now be used “out of the box” (Demko et al., 2024) and offer exciting potential for neuroscientific and clinical applications.

Despite progress, OPM-MEG remains a new technology and even the most advanced systems typically have fewer than 200 channels (Alem et al., 2023; Bonnet et al., 2025; Schofield et al., 2024; Tanner et al., 2025), whereas conventional instruments have closer to 300. (Here, our definition of a channel is an independent signal representing magnetic field (or magnetic field gradient) measured by the MEG system.) This lack of channels is important for three reasons. First, as sensors move closer to the head, field patterns become more focal (Boto et al., 2016; Iivanainen et al., 2017; Tierney et al., 2022); if the density of sensors is insufficient, then the largest magnetic fields can be missed (i.e., the field pattern is spatially aliased). This is particularly important for children, where the distance from the brain to the scalp surface (and sensors) is smaller than in adults (Boto et al., 2022) making field patterns even more focal. Second, in most MEG studies, fields from all channels are combined to create regional estimates of electrical activity in the brain—a process called source localisation. Increasing channel density affords an increase in the signal-to-noise ratio (SNR) of the reconstruction (Hill et al., 2024). Consequently, increasing channel count is key if OPM-MEG is to yield SNR values that are better than those achieved in conventional MEG. Finally, one significant advantage of OPMs is that their flexible placement allows coverage of regions that are hard to reach with conventional MEG—for example, cerebellum (Lin et al., 2019). However, extending coverage to these areas, whilst maintaining coverage of the whole cortex, requires higher sensor counts. It follows that if OPM-MEG is to realise its potential, we must develop systems with a larger number of channels.

Maintaining the synchronous operation of a large array of OPMs is challenging. From a technical point of view, each OPM is a complex system in which a vapour of alkali atoms is used to sense local magnetic field. To enable measurement, the quantum mechanical properties of the atoms must be altered, via both absorption of laser light (optical pumping; Happer, 1972) and controlled manipulation of the magnetic fields experienced by the vapour. This requires operation of a semiconductor laser and a set of electromagnetic coils inside the sensor (to generate the required fields). The thermal properties of the vapour must be tightly regulated (Allred et al., 2002) as must the frequency of the light generated by the laser (which is also a function of temperature). Additionally, the magnetometer signal is read out via measurement of the amplitude of laser light passing through the vapour, requiring control of a photodiode (and lock-in detection). Successful OPM-MEG, therefore, requires these control signals to be sent, and output signals received, independently yet synchronously, to and from each of the sensors in the array. This necessitates complex electronics, and the larger the array becomes, the larger the control system required.

Large arrays also pose significant practical problems. For example, source localisation requires that the locations of the OPMs and their sensitive orientations are known precisely. This is straightforward with small numbers of sensors since optical methods can be used to track sensor locations. However, it becomes difficult with high-density arrays, not only due to increased numbers of sensors and their proximity, but also because the sensors become obscured by cables. Source localisation also requires that the relationship between the output of a sensor (typically a voltage) and the magnetic field experienced by the sensor (the quantity required) is known accurately. This is termed the sensor gain and can vary from sensor to sensor (Hill et al., 2025); whilst it can be characterised by applying known fields to the sensor using on-board coils, running such an algorithm for many sensors can be time consuming. In addition, when sensors are in proximity, the field measurement made at one OPM can be affected by the presence of a second OPM (an effect known as crosstalk); this problem becomes worse when using high-density arrays and can alter both directional sensitivity and gain. For these reasons, fast techniques to determine sensor locations, orientations, and gains—accounting for cross-talk—(henceforth termed array calibration) are critical for high-density arrays to be effective. Finally, once the array is calibrated, techniques to ensure that the array captures a faithful record of the real magnetic fields present (i.e., quality assurance techniques) are required. This is important for all studies but becomes particularly critical if OPM-MEG systems are to be used clinically (e.g., for localisation of epileptogenic foci in pre-surgical mapping—a key application for high-density OPM-MEG systems; Feys et al., 2023; Rampp et al., 2020).

In this paper, we aim to construct a 384-channel OPM-MEG system. We demonstrate how synchronised integrated miniaturised electronic control systems (Schofield et al., 2024) can be used to control a large-scale array of OPMs. Further, we develop a novel calibration methodology (Hill et al., 2025) which can accurately determine the positions, orientations, and gains of the OPMs, and control for sensor crosstalk. We employ a “phantom” which generates known magnetic fields to perform quality assurance checks on our array. Finally, we demonstrate the system with human MEG data, via the characterisation of task-induced changes in neural oscillations during a visual task and the measurement of “baseline” oscillations during a naturalistic “movie-watching” task. We hypothesise that higher channel count will show advantages for both sensitivity to neuromagnetic effects and spatial resolution.

## 2 Methods

### 2.1 OPM-MEG system design

#### 2.1.1 Sensor array

Our sensor array was constructed using 128 “triaxial” sensors (QuSpin Zero Field Magnetometers, Colorado, USA), with each sensor able to measure magnetic fields in three orientations with equivalent sensitivity (Boto et al., 2022). These three measurements are independent, and thus 128 triaxial OPMs provide 384 channels of data.

Each OPM contains a cell housing with ^87^Rb vapour. Circularly polarised 795-nm light (resonant with the D1 transition for ^87^Rb atoms) is passed through the cell and photons are absorbed by the atoms, changing both their energy levels and angular momentum. The result is to align the atomic magnetic moments along the direction of the laser beam and introduce a bulk magnetisation into the vapour (Happer, 1972). Assuming no external magnetic field, the atoms become trapped in a single energy state; once in this state they can no longer absorb photons and the amount of laser light passing through the vapour is maximised. However, in the presence of an external magnetic field, the bulk magnetisation of the vapour interacts with the external field according to the Bloch equations; this changes the energy states occupied by individual atoms and those atoms can once again absorb photons, causing a drop in light transmission through the cell. This system could act as a magnetometer, however, the light passing through the cell (measured via a photodiode) is a Lorentzian function of magnetic field, peaking at zero. This makes it impossible to differentiate positive and negative fields. Thus, to add directional sensitivity, we use sinusoidally varying (923-Hz) modulation fields generated by on-board-sensor coils. This changes the solutions to the Bloch equations so sensor output (at the modulation frequency; measured via lock-in detection) becomes a linear function of the magnetic field through the cell, along the orientation of the modulation field (Cohen-Tannoudji et al., 1970) (i.e., if the laser beam is oriented in the z-direction and the modulation field is applied in x, we can measure the x-component of external field). It is possible to measure two field components perpendicular to the laser beam (the x and y components) simultaneously by providing two temporally and spatially orthogonal modulation fields (oriented along x and y). However, the sensor is much less sensitive to fields along the beam axis (z-direction). Thus, to build a triaxial sensor, two beams are passed through the cell at a relative angle of 90°. The first beam, oriented in z direction, allows field measurements in the x and y directions. The second beam, oriented in x direction, allows field measurements in the y and z directions. The two beams combined then provide characterisation of all three components of field (Boto et al., 2022; Shah et al., 2020) (with the two measurements in the y direction averaged). Importantly, to reach the high sensitivity required for MEG, the vapour must be heated to 150°C enabling operation in the spin exchange relaxation free (SERF) regime (Allred et al., 2002) which maintains coherence between atomic magnetic moments.

We chose to build our array using triaxial sensors due to three significant advantages: first, having three axes of field measurements (distinct from arrays with MEG sensitivity on a single axis (Alem et al., 2023; Hill et al., 2020) or two axes (Bonnet et al., 2025; Seedat et al., 2024)) maximises the density of channels over a given region of scalp. This, in turn, maximises the SNR for source reconstruction (Hill et al., 2024). Second, triaxial measurement has been shown to improve the differentiation of magnetic fields from the brain and fields generated in the environment (Brookes et al., 2021; Holmes et al., 2023). This is advantageous for interference rejection. Finally, triaxial measurement helps to improve the uniformity of coverage of the cortex (Boto et al., 2022), particularly for shallow areas and especially in babies and children. Each triaxial sensor was 12.4 × 16.6 × 24.4 mm^3^ and was mounted with its longer (24.4-mm) axis pointing radial to the scalp. Sensors had an expected noise floor of 10–20 fT/√Hz along all three axes.

**Fig. 1.**
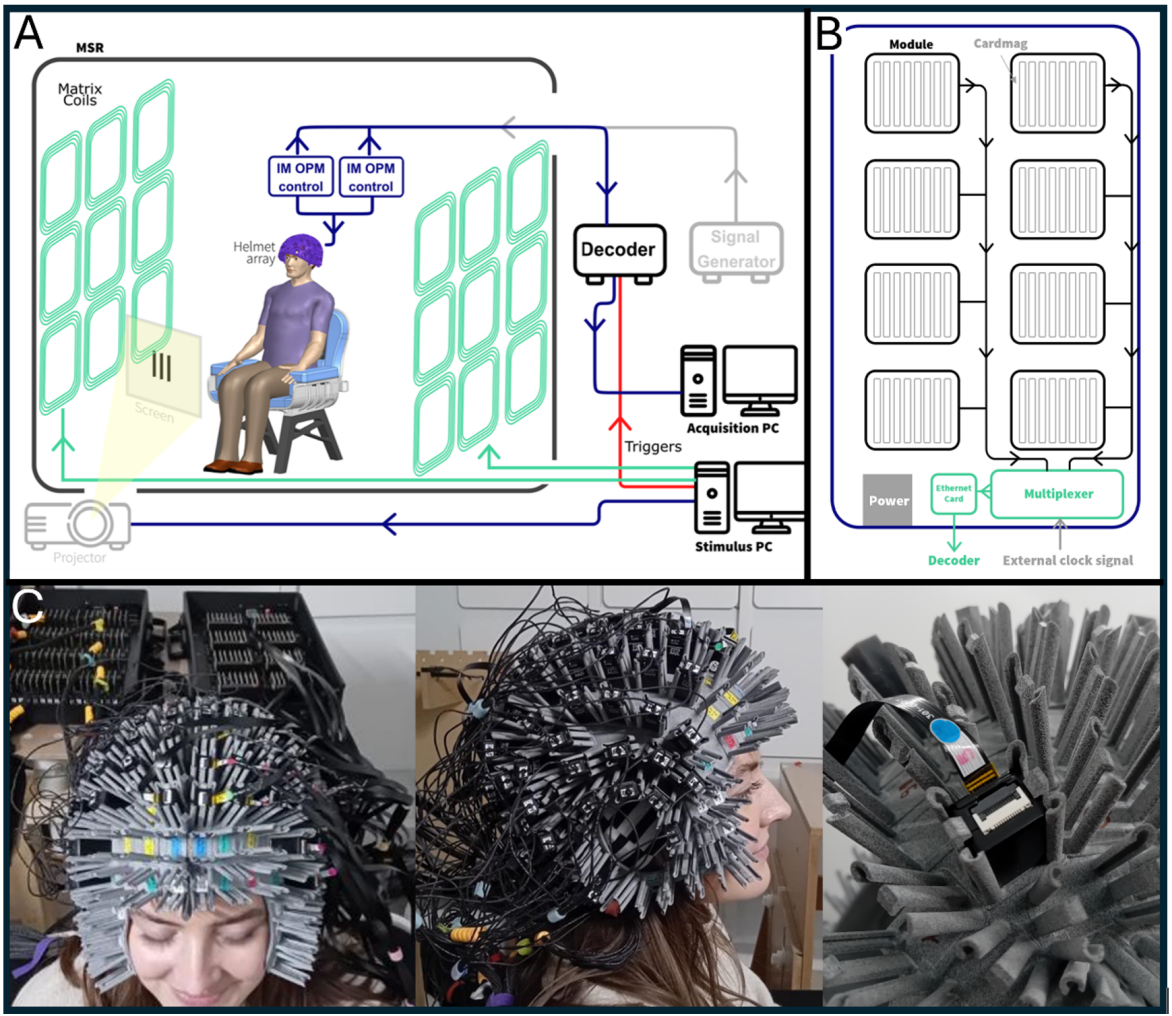
The OPM-MEG system. (A) Schematic diagram of the complete OPM-MEG system. (B) Schematic of an individual IM electronics control unit. (C) The 384-channel triaxial OPM-MEG array, with sensors mounted close to the scalp using a bespoke 3D-printed helmet. Each sensor is held on the helmet by a stanchion at each corner and two clips to prevent it sliding away from the scalp (right-hand panel).

The sensors were held on the scalp via a 3D-printed bespoke helmet (Cerca Magnetics Limited, Nottingham, UK) which was designed based on the subject’s MRI scan (Fig. 1C). The internal surface of the helmet was made to fit the subject’s scalp and face, whilst the external surface contained 163 approximately evenly spaced slots, into which the sensors could be located. The average distance from the scalp surface to the sensitive region of each sensor (i.e., the centre of the vapour cell, calculated for all available helmet slots) was 6.2 ± 5.0 mm (mean ± standard deviation). Because the helmet was based on the subject’s MRI, the locations of the sensor casings relative to brain anatomy were known a-priori from the computer aided design (CAD) files of the helmet.

#### 2.1.2 Electronics

The sensors were controlled by two integrated miniaturised electronics units (Schofield et al., 2024; *Neuro-1-electronics*, QuSpin, Colorado, USA—https://quspin.com/neuro-1-an-integrated-sensor-system-for-opm-meg/) working in concert, with each unit controlling 64 OPMs.

Within a single unit, each OPM is controlled by a separate circuit board (a “cardmag”), which provides control signals for all on-board sensor components and enables lock-in detection to read the output signals. Four signals are initially read from the two photodiodes in the sensor (these reflect fields in the x, y (measured twice), and z orientations). The cardmag then outputs three *digital* signals (which are directly proportional to magnetic fields along the three sensor axes). Cardmags are grouped together in “modules”, with each module containing eight cardmags. Each electronics unit houses 8 modules to control a total of 64 sensors. The outputs from each module are combined using a multiplexer and sent to a network card. Each electronics unit is 0.36 × 0.2 × 0.06 m^3^ and weighs 0.81 kg. Importantly, to make the two independent units work together, they require a synchronous clock. This was provided by a signal generator which made a 12.288-MHz, 1.8-V (peak-to-peak, positive voltage only) sine wave, and was fed to both units. In theory, it would be possible to combine any number of units/modules/cards in this way so the total channel count could be increased (or decreased) in accordance with requirements for any MEG experiment.

Both electronics units were connected via Ethernet to a data acquisition system (DAQ) based on a FPGA controller (sbRIO9637, National Instruments), which integrates the MEG signals from all 384 channels and combines them with external inputs representing stimulus timing (termed “triggers”). All signals are then synchronously sampled and passed to an acquisition PC via an ethernet connection for visualisation and storage. A schematic illustration of the system is shown in Figure 1A, along with a schematic of the individual control units in Figure 1B.

As an aside, the two units are also capable of three-axis closed-loop operation (in which the magnetic field is measured at each OPM along all three axes, and the electronics effects a negative feedback loop, whereby currents are applied to the on-board sensor coils to maintain zero field at the vapour cell). This has been shown to linearise the OPM response in cases where background field is high (Schofield et al., 2024). However, this was not employed for the current work as large dynamic range was not required. In addition, the decoder also has 16 analogue-to-digital converter channels and 9 digital inputs. These auxiliary channels can be used to sample additional signals synchronously with OPM data (e.g., signals from stimulus equipment).

#### 2.1.3 Magnetic shielding

The sensor array and electronics units were housed inside a magnetically shielded room (MSR) comprising four MuMetal layers to attenuate DC/low frequency and one copper layer to attenuate high-frequency magnetic interference fields (MuRoom, Magnetic Shields Limited, Kent, UK). The MSR walls were equipped with degaussing coils to reduce remnant magnetisation in the MuMetal prior to a scan. The MSR was also equipped with a matrix coil system (Holmes et al., 2023) capable of active field control (Cerca Magnetics Limited, Nottingham, UK). Active field compensation was not used for these experiments. However, the matrix coil was used to provide fields for external calibration (see below).

#### 2.1.4 System control

A single “acquisition” computer was used for OPM-MEG control, degaussing, and data acquisition; the experimental paradigms (along with associated triggers), phantom control, and matrix coil calibration procedures (see below) were controlled by a second “stimulus” computer. The MSR was equipped with a ViewSonic PX748-4K projector (refresh rate 120 Hz) to present visual stimuli into the MSR, via projection through a waveguide onto a back projection screen positioned ~100 cm in front of the subject.

### 2.2 System calibration

System calibration involves finding of the locations of the sensors (relative to each other), the orientations of their sensitive axes and the gain of each channel. We did this by imposing a set of known magnetic fields onto the array, and fitting a model of those fields to the measurements made at the sensors (Hill et al., 2025; Iivanainen et al., 2022).

To generate the fields, we used the matrix coil which comprised 96 independently controllable coil elements (Holmes et al., 2023) spaced approximately evenly on all 6 faces of the MSR. The field per unit current generated by each coil element had previously been mapped throughout the centre of the MSR using a fluxgate magnetometer (Hill et al., 2025). Using this mapping, we determined the optimum combination of coil currents to create three uniform fields oriented in x ($B_{x}$), y ($B_{y}$), and z ($B_{z}$). In addition, we determined the optimum combination of coil elements to generate the five independent magnetic field gradients; $\frac{dB_{x}}{dx}$ , $\frac{dB_{x}}{dy}$ , $\frac{dB_{x}}{dz}$ , $\frac{dB_{z}}{dz}$ , $\frac{dB_{y}}{dz}$ .

The coils were operated so each of the eight fields were generated simultaneously, by currents fed to the matrix coils at different frequencies (3–10 Hz in steps of 1 Hz) (i.e., a field oscillating a 3 Hz represented $B_{x}$, a field at 4 Hz represented $B_{y}$ and so on). We generated all eight fields for a period of 4 s. We then took a segment of data and, for each channel, used a Fourier transform to quantify the direction and magnitude of the measured field at each of the eight frequencies.

We used the three uniform fields to determine the orientation and gain of each channel. Specifically, we modelled the signal measured by a single channel as

$$\text{b}={\text{B}}_{\text{u}}\text{g}.$$ (1)

Here, ***b*** represents a three-element column vector corresponding to the magnitude of the three uniform field components, measured by a single channel. g represents the (unknown) sensor gain along three orthogonal axes and ${\text{B}}_{\text{u}}$ is a diagonal square matrix whose diagonal elements represent the known magnitude of the three uniform field components ($B_{x}$, $B_{y},$ and $B_{z}$) generated by the matrix coil, which were set to 0.2 nT along the x, y, and z directions. We then solved the equation for g ($={\text{B}}_{\text{u}}^{+}\text{b}$ ; where ${\text{B}}_{\text{u}}^{+}$ is the pseudo-inverse of ${\text{B}}_{\text{u}}$); normalisation of g gives channel orientation, and its magnitude gives the channel gain.

Following this, we used the five field gradient terms, alongside knowledge of the channel orientation and gain, to solve for the channel location. Specifically, we can write

$${\text{b}}_{\text{g}}={\text{B}}_{\text{G}}\text{r},$$ (2)

where $b_{g}$ is the five-element column vector representing the signal measured at each channel due to each of the five gradients. $B_{G}$ is a 5 × 3 matrix, where a single column characterises the vector components of field that would be measured, based on the estimated channel orientation and gain, if the channel were at a distance of 1-m from the origin of the coordinate system (the centre of the helmet) in all three cartesian directions. (i.e., column 1 represents the fields for a $\frac{dB_{x}}{dx}$ gradient; column 2 the $\frac{dB_{x}}{dy}$ gradient, and so on.) Multiplying this matrix by the three-element column vector r (which represents the channel location) generates a model of the measured gradient fields. To solve for r (i.e., to obtain the channel location), we again use the pseudo-inverse approach, computing ${\text{B}}_{\text{G}}^{+}$. For each sensor (i.e., three orthogonal channels), we took the position to be the average of the three channel positions.

The amplitudes of the field components (0.2 nT for the uniform fields and 2 nT/m for the gradients) were chosen such that their superposition (at any point in time) did not exceed 1 nT total amplitude (this ensured that the OPMs (operating in open loop) maintained their linear regime). These amplitudes can be generated accurately using the matrix coil system electronics (which has a least significant bit field size of 1 pT); the SNR of the responses was also high, given the sensor noise floor of ~15–20 fT/√Hz. By repeating this process for every channel (sequentially), we derive a complete description of the locations, orientations, and gains of every channel in the array (in the coordinate system of the matrix coil).

This same approach (with 18 coil elements) was used to produce a starting guess for a more advanced spherical harmonic refinement (based on matching channel responses to spherical harmonic models of the fields produced by each coil) (Iivanainen et al., 2022). Here, the larger number of coil elements ostensibly enables more accurate generation of field variations and removes the need for the harmonic refinement. Our own previous work (Hill et al., 2025) used a “coil-by-coil” approach (whereby signals from 96 coils were fitted independently). However, this required 20-s of data to spectrally resolve signals from all 96 coil elements. Here, by condensing to just eight signals, the acquisition time can be dramatically reduced (see also Appendix A1). Importantly, this calibration can be performed with the helmet in situ on the subject’s head, meaning it can be adapted for use in, for example, flexible (EEG-cap-style) helmets.

### 2.3 Phantom experiments

To test the accuracy of our 384-channel array (and its calibration), we used an electromagnetic phantom (Cerca Magnetics Limited, Nottingham, UK) which generates controlled and well characterised magnetic fields. The phantom was constructed from a semi-circular printed circuit board (PCB) containing five magnetic dipoles. Each dipole comprised a spiral coil fabricated on a two-layer PCB, with 20 turns on each layer connected by a via at the spiral’s centre. The spiral’s inner radius was 1.4 mm with an outer radius of 6.3 mm. The dipoles are positioned on the PCB along a circular path, at a radius of 7.5 cm, at intervals of 30°. Each dipole is supplied with current via wire paths on the PCB connected to a twisted pair cable, which runs from the phantom to the outside of the MSR. This was connected to a voltage source (National Instruments NI-9264 16-bit digital-to-analogue converter) with a 56-kΩ resistor in series and controlled using MATLAB.

The phantom was placed at the centre of the MSR inside a 3D-printed helmet with the dipoles running approximately left to right. (Note, the helmet used to accommodate the phantom was slightly larger than that used for the human study, but similar in design (see Fig. 3A).) Each dipole was energised sequentially: within a single “trial”, the dipole was energised for 2 s using an oscillating driving voltage at 27 Hz, with a peak-to-peak amplitude of 1 V. This was followed by a 1-s window with the dipole switched off. We repeated 50 trials of the experiment for each dipole (total experimental time 150 s). Data were recorded from all OPM channels throughout. This process was repeated for all five dipoles. We repeated the whole experiment twice.

#### 2.3.1 Phantom data analysis

Data were segmented and averaged across trials for each dipole. Within the average trial, there was a 2-s window with the dipole switched on and oscillating at 27 Hz. This meant there were 108 independent time points at which there was a maxima or minima in measured field. From these we selected 100 time points, omitting the first and last 4 peaks. We then used a dipole fitting algorithm to determine the location, orientation, and amplitude of the dipole; these values were derived for each time point independently (i.e., 100 independent dipole fits). Models were created based on the equation for the field produced by a magnetic dipole and fitted using non-linear regression (specifically the *nlinfit* function in MATLAB, which minimises the summed squared difference between the measured and modelled fields). This was repeated for each of the 5 dipoles, and for both repeats of the experiment, resulting in 200 localisations for each dipole. We then undertook two analyses to determine the accuracy of the result:

1. **Relative error in dipole position**: Having determined the locations of the 5 dipoles, we computed the distances between all possible dipole pairs (there are 10 measurable distances between locations). We then compared these distances with their known values from the PCB manufacturing process (using the centre of the spirals). Note that this only provides a measure of *relative error* between dipole locations and not absolute error in dipole position; the latter not being possible with the present phantom since its absolute position with respect to the sensor array was unknown.
2. **Correlation between measured and modelled fields**: For each of the five dipoles, we measured the Pearson correlation coefficient between the measured and modelled magnetic field patterns.

The above analyses were repeated twice; once where we used the sensor locations, orientations, and gains derived using calibration, and a second time where sensor locations and orientations were taken directly from the computer aided design (CAD) file for the helmet—this assumes that channel orientations are orthogonal and parallel to the outer casing of the sensor, and that all sensor gains were unity. We hypothesised that distance errors would be lower and correlations higher when using calibration.

### 2.4 Human experiments

#### 2.4.1 Experimental paradigms and data recording

To test the suitability of our system for recording human MEG data, a single participant (who had previous experience participating in MEG studies) undertook a series of experiments. The participant provided written informed consent prior to taking part, and all experiments were approved by the University of Nottingham School of Medicine and Health Sciences Research Ethics Committee. We used two experimental paradigms:

- **Visual experiment:** Visual stimulation was applied using a circular grating, displayed centrally at a visual angle of 24°, with 3 cycles per degree. A single trial comprised 2 s of stimulation where the circular grating was static, followed by 2 s where the grating was allowed to drift inwards at a rate of 2°s^-1^. This was followed by a rest period of 6 s during which a white fixation cross was located centrally on a black screen. Sixty trials were shown, making the total experimental duration 600 s. Triggers were sent from the stimulus PC to the OPM-MEG decoder via a parallel port. On cessation of visual stimulation, the subject was asked to press a button with the index finger of their right hand. This stimulus generates both a task-induced reduction in alpha oscillations and an increase in gamma oscillations, in visual cortex (Fries et al., 2008).
- **Movie-watching experiment:** The participant watched a clip from the film “Dog Day Afternoon” (also used in Rier et al., 2023). Data were recorded for 622 ± 8 s (mean ± standard deviation across runs). Here, we aimed to characterise oscillatory power in all brain regions.

During both experiments, the participant was free to move but not told to do so. In a single recording session, the participant was first taken into the MSR and sat comfortably on a support at the centre of the room, wearing the bespoke helmet (Fig. 1C). Following this, the MSR door was closed and its walls degaussed, using a procedure that takes ~1 min. System calibration was then carried out (with the helmet in situ on the subject’s head). The two experimental paradigms were then run, with OPM-MEG data recorded at a sample rate of 375 Hz. The session was repeated five times.

Following collection, all data were organised according to the Brain Imaging Data Structure (BIDS) for MEG (Niso et al., 2018). The participant also had an anatomical MRI scan (1-mm isotropic spatial resolution; T1 contrast; 3 T Phillips Ingenia scanner). This was acquired beforehand and used to create the helmet.

#### 2.4.2 Visual task: Data preprocessing

All data processing for the visual task was carried out using the Fieldtrip toolbox (Oostenveld et al., 2011) alongside custom written code in MATLAB. A notch filter (50 Hz + harmonics) was applied to all OPM-MEG data to remove powerline noise. Data were then bandpass filtered into the 1–150 Hz band using a 4th-order Butterworth filter. A power spectral density (PSD) plot was created using a technique similar to Welch’s method (Welch, 1967); data were segmented into 10-s chunks using a flat-top window, the power spectral density was calculated for each data chunk (using MATLAB’s periodogram function) and the resulting spectra (for all chunks) were averaged. PSD was derived for all channels, and any channels with no signal (<7 fT/√(Hz)) or showing high noise (>40 fT/√(Hz)) in the 60–80-Hz range were removed (based on visual inspection). Homogeneous field correction (HFC) (Tierney et al., 2021) was applied to remove interference that manifests as a spatially uniform field across the OPM array. OPM-MEG data recorded were segmented into 10-s trials (0 s < t < 10 s relative to visual stimulus onset). All trials were inspected visually and any trials with high noise levels were removed.

#### 2.4.3 Visual task: Gamma band oscillations

Data were processed using beamforming (Robinson & Vrba, 1998), an established technique which works well for localisation of neural oscillatory effects (Brookes et al., 2008). We constructed images showing the spatial signature of task-induced gamma band power change. Data were filtered to the 40–60-Hz band and a data covariance matrix was constructed using data recorded throughout the whole experiment (excluding bad trials). Tikhonov regularisation was applied to the matrix (using a regularisation parameter equal to 1% of the maximum eigenvalue of the unregularised matrix). The brain volume was divided into voxels on a 4-mm cubic grid and the beamformer weighting parameters (which define the linear combination of channels that optimally describe the electrophysiological signal at each voxel) were constructed, using the regularised data covariance matrix; the forward model (i.e., the model of magnetic fields generated at all sensors by an equivalent current dipole located at each voxel (with a predetermined orientation)) was based on a single shell volume conductor model (Nolte, 2003). To make a “pseudo-T-statistical” image, we contrasted projected oscillatory power in the 2-s to 4-s (active) window to oscillatory power in the 7-s to 9-s (control) window (timings relative to onset of visual stimulation). Pseudo-T-statistics were derived for all voxels, with source orientation determined as the direction of maximum beamformer projected signal power (Sekihara et al., 2004). The resulting Pseudo-T-statistical images were then averaged across the five experimental runs.

For the location showing the largest gamma response (derived individually for each experimental run), we generated a time–frequency spectrogram (TFS). Here, beamformer weights were re-derived using a covariance matrix calculated in the 1–150 Hz band and regularised as above (Note: the narrower (40–60 Hz) frequency range was used for the pseudo-T-statistical image to maximise spatial resolution; this was expanded for the TFS since we wanted to examine activity at a single location across a wider range, and this requires a matched frequency window for the weights calculation; Brookes et al., 2008). We again used a single shell volume conductor for the forward model. These weights were used to derive a broadband estimate of the time course of electrophysiological activity at the location of interest (i.e., a virtual electrode (VE)). VE data were then frequency filtered into overlapping bands between 1 Hz and 120 Hz (specifically: 1–4 Hz; 2–6 Hz; 4–8 Hz; 6–10 Hz; 8–13 Hz; 10–20 Hz and then 10-Hz wide bands, overlapping by 5 Hz, (i.e., 15–25 Hz; 20–30 Hz; etc.) up to 120 Hz). For each band, a Hilbert transform was used to derive the analytic signal, and the absolute value of the analytic signal was calculated to generate the instantaneous amplitude of the band-limited oscillations—termed the Hilbert envelope. This was averaged over trials and concatenated across frequencies. For all frequency bands, we calculated a baseline oscillatory amplitude (in the 9 s ≤ t ≤ 10 s time window). We then computed the final TFS as the difference between instantaneous oscillatory amplitude and baseline, normalised by the baseline to give relative change in oscillatory amplitude.

In addition to the TFS, we derived the trial-averaged gamma band Hilbert envelope in the 40 to 60 Hz frequency range (using band limited covariance and beamformer weights). We then computed SNR as the mean difference in Hilbert envelope between the active and rest windows, divided by standard deviation of the envelope in the rest window.

All of the above analyses were carried out using the full sensor array and also using a subset of approximately half the sensors (equidistantly located across the scalp) to understand the effect of channel count.

#### 2.4.4 Visual task: SNR and the effect of high channel count

To further investigate how channel density affects SNR, we also computed the gamma envelope with a random selection of sensors in the array excluded from the analysis. By removing sensors, the number of channels used was allowed to vary from 1 to 331, in steps of 10, with 20 iterations of random sensor distributions for each channel count. To assess the effect of varying the number of channels, we plotted SNR as a function of the total measurable signal, which was quantified as the Frobenius norm of the forward model vector, $\left\|\;l\;\right\|=\sqrt{\sum_{\text{i}=1}^{\text{N}}{\text{l}}_{\text{i}}^{2}}$ (where ${\text{l}}_{\text{i}}$ is the forward model for channel i and N is the total number of channels used). Note that ‖ l ‖ changes with the square root of the total channel count (Hill et al., 2024). For these calculations, we computed a gamma envelope with no HFC applied to allow the full range of channel counts to be used. (At the lower end of the range, there would not be sufficient channels to perform HFC.)

#### 2.4.5 Movie-watching task: Preprocessing

Notch filters were applied at the powerline frequency (50 Hz + harmonics), and data were filtered into the 5–150 Hz band. Then, an automated outlier detection procedure, adapted from OSL-ephys (van Es et al., 2025), was used to identify and remove channels and data segments with either zero signal or high noise. Specifically, channels with standard deviations close to 0 were removed, and we used an algorithm to detect outliers in channel variance via a “generalised extreme studentised deviate” (GESD) approach (Rosner, 1983). Bad segments of data were also found by detecting outliers (using GESD) in standard deviation, calculated for non-overlapping 1000-sample (2.67 s) long segments. Following preprocessing, data processing for the movie-watching task was carried out using MNE-python (Gramfort et al., 2013).

#### 2.4.6 Movie-watching task: Spectral power

We parcellated the brain into distinct anatomical regions according to the Automated Anatomical Labelling (AAL) atlas. This was achieved by co-registering the AAL atlas to the participants anatomical MRI scan, using FLIRT in FSL (Jenkinson & Smith, 2001; Jenkinson et al., 2002). We found the location of the medoid voxel of each AAL region and generated a VE time course at those locations. To calculate the VEs, beamformer weights were derived using data covariance computed in the 5–150 Hz band, and a time window encompassing the entire experiment (excluding bad data segments). The forward model was based on an equivalent current dipole and a single-shell volume conductor (Nolte, 2003). The covariance matrix was regularised, using a regularisation parameter equal to 5% of the maximum eigenvalue of the unregularized matrix. The beamformer weights were normalised to account for depth bias (Robinson & Vrba 1998). This process resulted in 78 time courses, representing each of the AAL regions.

For each regional time course, we took the broadband (5–150 Hz) beamformer projected data, normalised by its standard deviation. We then frequency filtered these normalised VE data to both the alpha (8–13 Hz) and beta (13–30 Hz) bands (using a 4th-order, zero-phase-shift Butterworth filter). We then calculated the variance of the filtered data (i.e., the alpha and beta band variance) and (because the original data are normalised) this gives a measure of “relative alpha/beta power” (i.e., the fraction of the total variance that exists in the alpha or beta band) (Rier et al., 2024). The calculation was repeated for all brain regions and the relative alpha/beta power plotted as functional maps. We hypothesised that, similar to the literature (Hunt et al., 2016), relative alpha power would peak in the occipital regions whilst relative beta power would be a maximum in the parietal lobes and sensorimotor regions. In addition, we also derived the Amplitude Spectral Density (ASD) for each region as the square root of the PSD (computed as above).

#### 2.4.7 Movie-watching task: Spatial resolution

Finally, we aimed to assess the effect of high channel count on our movie-watching data, reasoning that increasing the number of channels would improve spatial resolution. In this context, lower spatial resolution means higher leakage of signal between the time courses for each AAL region. To quantify this, for every possible pair of brain regions in the AAL parcellation, we quantified zero-phase-lag signal leakage between locations using linear regression (Brookes et al., 2012) (this effectively measures the shared variance between brain locations, which becomes larger for lower spatial resolution). This was plotted as a matrix, where the element [i,j] represents the source leakage between each pair of regions, i and j. We then averaged these matrices across columns, yielding a 78-element vector whose elements represent the total leakage from a single region to all other regions. Having computed this for the data reconstructed using the full (384-channel) array, we then randomly selected half the channels and recomputed the VE time courses, and the leakage. This process was repeated 20 times to obtain multiple realisations of the (randomised) reduced-channel array. We hypothesised that leakage would be lower for the 384-channel array compared with the reduced-channel arrays.

## 3 Results

### 3.1 System performance and calibration

**Fig. 2.**
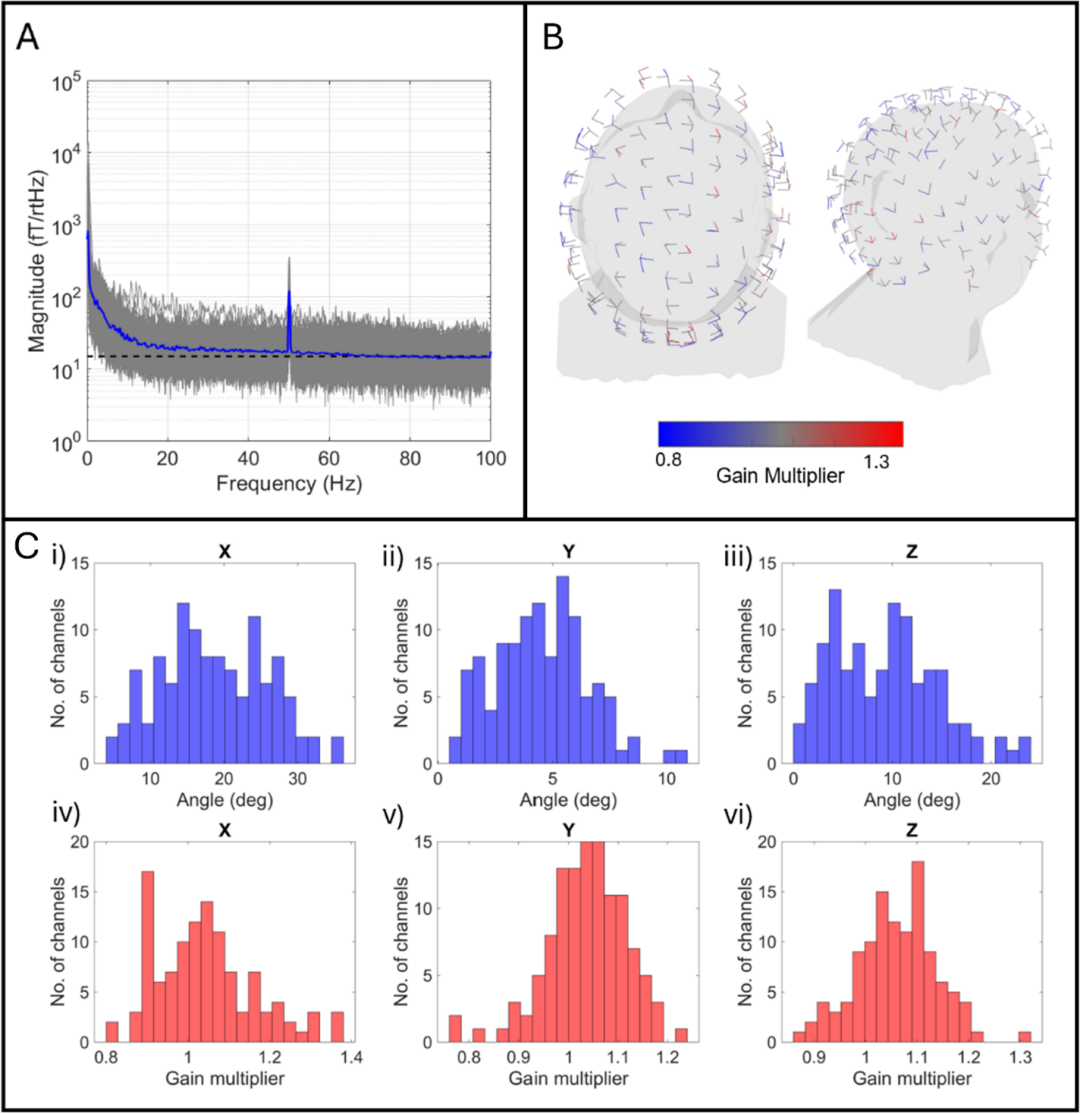
Sensor performance and calibration. (A) Power spectral density of empty room noise, measured for 346 channels and overlaid. The dashed line is at 15 fT/sqrt(Hz), and the blue line shows the average of all channels. Data shown after application of HFC. (B) Calibration-derived channel locations, orientations, and gains. Each channel is represented by an arrow which denotes the sensitive orientation. The point where multiple arrows converge is the location of the sensitive volume of a sensor. Channel gains are presented by the colour of the arrow. (C) The upper row (i–iii) shows the angle difference between the calibration-derived channel orientation and that expected from the CAD of the helmet. The lower row (iv–vi) shows the calibration-derived gains (relative to the factory-derived gain of one).

Figure 2A shows the noise level of the sensors when operated (in an empty room) as a high channel count system. Power spectral density is overlaid for all channels, and the dashed black line represents a 15 fT/√Hz level. Note that we removed 38 channels, either due to them measuring no signal, or having high noise. Of the remaining 346 sensors, the median noise in the 3 to 100 Hz frequency band was 17.0 fT/√Hz. This is marginally higher than previous reports for triaxial sensors. For example, Boto et al. (2022) reported a range of 9.9 fT/√Hz to 14.9 fT/√Hz (depending on which axis was measured) in the 10–90 Hz range; Schofield et al. (2024) reported 14 fT/√Hz (in the 3–100 Hz range) using a 192-channel array. The reason for the higher median here is likely due to our inclusion of some older (prototype) sensors being included in our array. It is noteworthy that this triaxial noise floor differs from that reported for dual or single-axis sensors (e.g., Boto et al. (2022) report 11.3 ± 1.5 fT/√Hz (y axis) and 13.8 ± 1.1 fT/√Hz (z-axis) for dual-axis sensors, whilst Alem et al. (2023) report 20 ± 5 fT/√Hz for radial sensors).

Figure 2B shows the channel layout derived using the calibration process. The direction of each arrow represents the channel orientation; the locations where three arrows converge represent the sensor locations, and the colour represents the calibration-derived gain value. In Figure 2C, panels i–iii show histograms (computed across all channels) of the angles between the calibration-derived channel orientation and the orientation of the sensor casing (according to the CAD file of the helmet). (I.e., an angle of 10° means the orientation of the sensitive OPM axis differs by 10° from the orientation of its outer casing.) Note that we separate out angles for the x-, y-, and z-axis of each sensor. Whilst the sensitive axes broadly point along three axes of the sensor casing, there are differences of 18.7 ± 7.3° for X-axes, 4.5 ± 2.0° for Y-axes, and 9.3 ± 5.4° for Z-axes. This is in close agreement with previous results (x-axis: 17.2 ± 8.5°, y-axis: 5.4 ± 2.4°, z-axis: 10.5 ± 5.4°) using a similar system (with fewer sensors). Zetter et al. (2018) found that channel orientation should be known to a precision of <10° for MEG reconstruction; this would be met by most (but not all) channels using the CAD determined orientations but would be met (and bettered) in full when using calibration-derived orientations.

Finally, Figure 2C—panels iv–vi shows histograms of the calibration-derived gain values for all sensors, again the x-, y-, and z-axis are shown separately. Here a value of 1 would indicate perfect agreement with the factory-derived gain. The true gain values vary between 0.8 and 1.4, with mean values of 1.0 ± 0.1 for X-axes, 1.0 ± 0.1 for Y-axes, and 1.1 ± 0.1 for Z-axes, in agreement with previous findings (Hill et al., 2025).

### 3.2 Phantom data

**Fig. 3.**
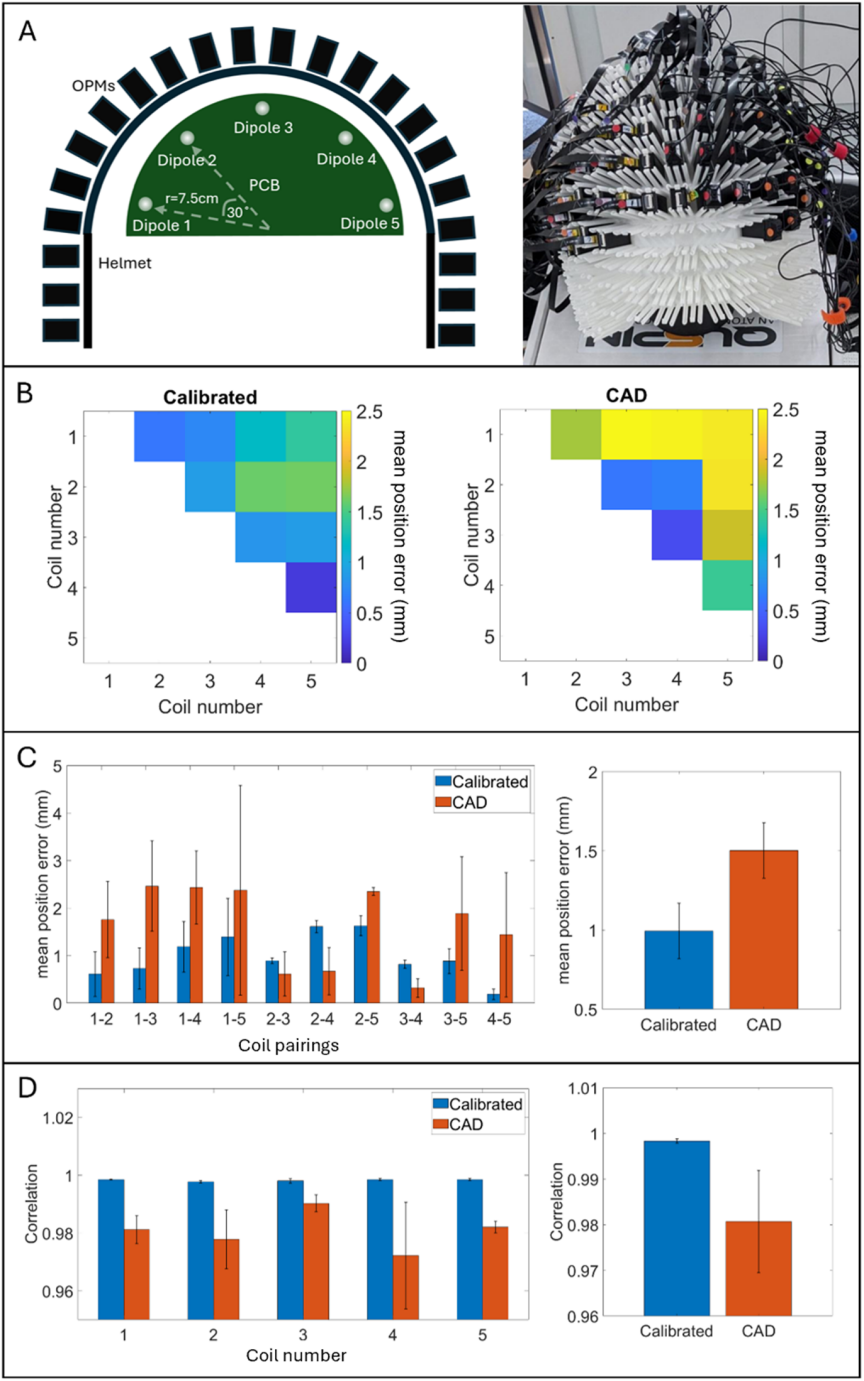
Phantom results. (A) The left panel shows a schematic of the phantom positioned in the helmet, with magnetic dipoles running (approximately) left to right. The right panel shows a photograph of the experimental set up. (B) Matrices show relative error in dipole location: that is, the difference in the distance between dipole pairs measured using dipole fitting, and the PCB manufacture (the ground truth). Left panel shows with calibration and right panel shows without calibration. Data averaged across 200 dipole localisations. (C) The left panel shows the same data as (B) plotted as a bar chart with the error bars showing standard deviation across all 200 localisations. The right panel shows relative errors, collapsed across all dipole pairs with the error bar representing standard deviation across dipole pairs. (D) Correlation between the recorded fields and the magnetic dipole model. Left panel shows correlation values for all 5 dipoles independently; error bars show standard deviation over 200 dipole fits for each dipole. Right panel shows correlation values averaged across dipoles, with error bars representing standard deviation between dipoles.

Figure 3 shows the experimental setup (panel A) and results (panels B–D) of our phantom study. To quantify the error in localisation, recall we measured the distances between all possible pairs of fitted magnetic dipole locations and compared the results with the equivalent distances measured via the PCB manufacture (the latter representing a “ground truth”). These relative errors in dipole position are shown as matrices in Figure 3B (e.g., element [1,2] represents the relative error between dipole 1 and dipole 2). Results were derived with (left) and without (right) calibration; data are shown averaged over 200 localisations for each dipole. These relative errors in position are further quantified in the left panel of Figure 3C, which shows the same data, unpacked into a bar chart (i.e., “1–2” represents the error between dipoles 1 and 2). Here, the error bars on the graph show standard deviation across all 200 dipole localisations; the red bars show data with calibration and blue bars show data without calibration. The right panel shows the same data, collapsed across all dipole pairs, and the error bar represents standard deviation across dipole pairs. With calibration, the average relative error in dipole position was ~1 mm, but this increased to >1.5 mm without calibration.

Figure 3D shows the correlations between the magnetic field patterns measured by the OPM array, and the fields predicted by a magnetic dipole model (following the dipole fit). In the left panel, correlations for all 5 dipoles are shown, with error bars representing standard deviation across 200 dipole localisations. The right panel shows the average correlation values across all dipoles, with error bars representing standard deviation across dipoles. With calibration, the average correlation is >0.998 but without calibration, this drops to <0.98. Both the relative error in dipole position and correlation demonstrate that (1) the OPM-MEG system is capable of localising magnetic dipoles with high accuracy and (2) there are advantages to calibration.

### 3.3 Human experiments: Visual gamma results

**Fig. 4.**
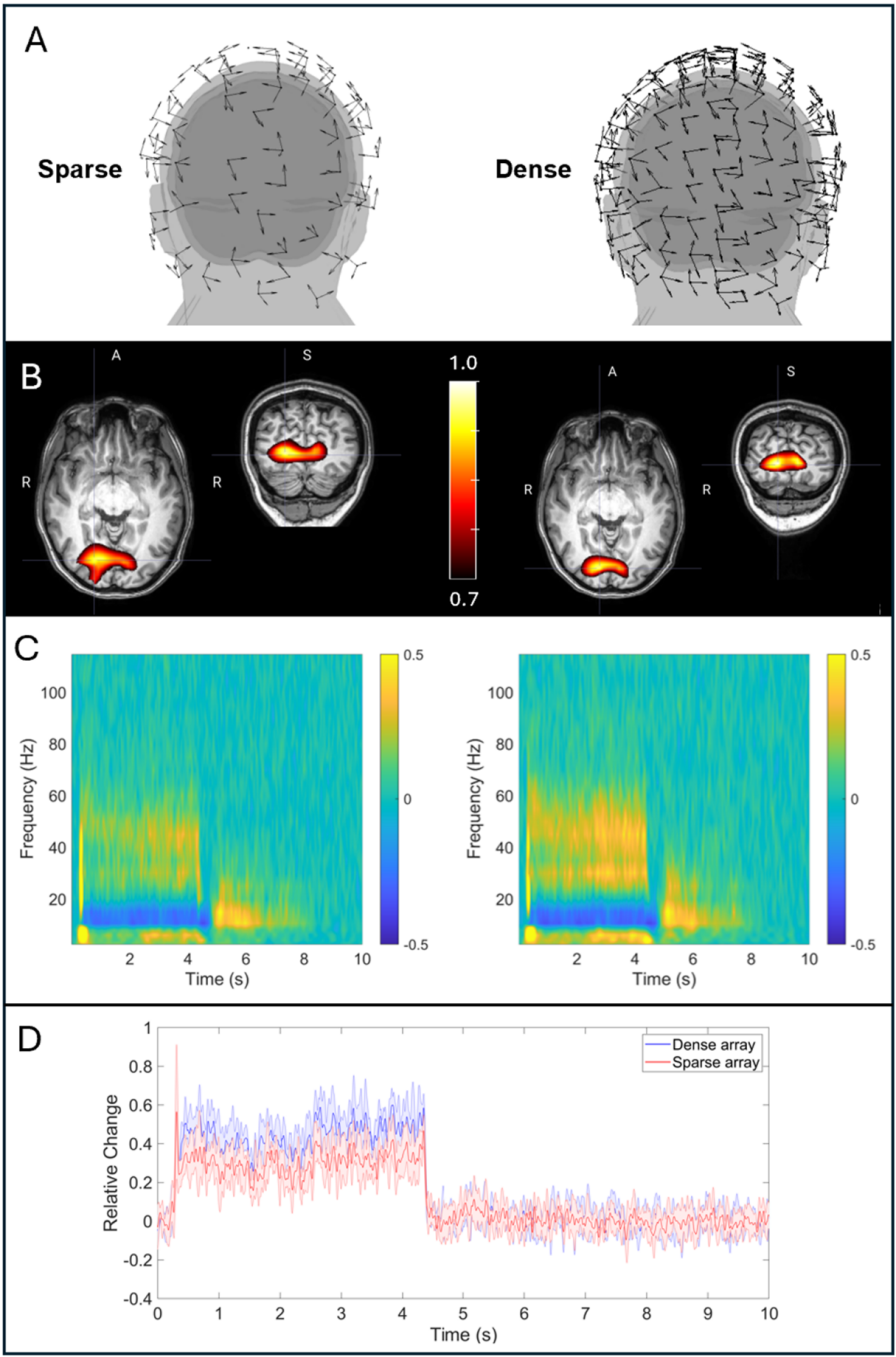
Visual gamma results. (A) Example sensor layouts for the sparse (left) and dense (right) arrays. (B) Spatial distribution of gamma power change with visual stimulation (shown in red/yellow) overlaid onto the subject’s anatomical MRI. Data have been averaged across five experimental repeats. (C) TFS data from the location of maximum stimulus-induced gamma power change. The locations were chosen independently for each experimental repeat and for each array; results are averaged across repeats. In both (B) and (C), data for the sparse array are shown on the left and the dense array on the right. (D) Line plots showing change in gamma amplitude at the location of maximum gamma power change. Locations are again chosen independently for each run and both arrays and data are averaged across five experimental runs. The shaded regions show standard deviation across repeats. Data for the sparse array are shown in red and the dense array in blue.

Figure 4 shows results from the visual experiment. On average, across the 5 runs of the experiment, we had 349 ± 3 working channels. We removed 6.2 ± 0.7 trials due to artefacts in the data.

Figure 4A shows representative examples of the arrays used, on the left the “sparse” array (165 ± 3 channels, representing a subset of approximately evenly distributed sensors after bad channel removal) and on the right the dense array (349 ± 3 channels). Figure 4B shows the spatial signature of stimulus-induced change in gamma power, plotted in red/yellow and overlaid on the subjects MRI. The left image shows the case for the sparse array, and the right image shows the dense array. Both images are averaged across five experimental repeats. In both cases, the location of the largest gamma change is in primary visual areas extending to both hemispheres, which is consistent with a centrally presented visual stimulus. When thresholding the two images (at 70% of their maximum value), the volume of the remaining peak decreased from 18.8 ± 9.2 cm^3^ (mean and standard deviation across runs) for the sparse array to 16.7 ± 6.6 cm^3^ for the dense array, indicating a slight improvement in spatial specificity with increased channel count.

Figure 4C shows average TFS representations of the VE data extracted from the location of largest gamma response. Again, the left panel shows the sparse array, and the right panel shows the dense array; yellow represents an increase in oscillatory amplitude relative to baseline; blue represents a decrease. In both cases, an increase in gamma oscillations at approximately 45 Hz is apparent and this is qualitatively consistent with the literature using similar paradigms (e.g., Fries et al., 2008). The line-plots in Figure 4D show the gamma Hilbert envelope, averaged across experimental runs (with standard deviation shown by the shaded area). Quantitatively, for the dense array (blue line) the increase in gamma amplitude was 49 ± 6% compared with baseline (mean and standard deviation across experimental runs, measured in the 0.5–4 s window). It is challenging to compare this with similar metrics in the literature since gamma amplitude varies between individuals (Muthukumaraswamy et al., 2010), and here we only scanned a single participant. Nevertheless, a similar paradigm (Rhodes et al., 2025) generated gamma increases between 20% and 50% (at the group level, in adults) using 192-channel OPM-MEG; a study using conventional MEG reported changes ranging from 5% to 40% (Muthukumaraswamy et al., 2010) (albeit with a different stimulus). The gamma change measured here is, therefore, consistent with this literature, though direct comparisons should be treated with caution due to inter-individual differences and the impact of paradigm design.

Most importantly, by reducing the channel count from 349 ± 3 to 165 ± 3, the relative change in gamma amplitude drops from 49 ± 6% (blue line in Fig. 4D) to 32 ± 9% (red line). The total possible measurable signal (quantified as the Frobenius norm of the forward model from the location of maximum gamma change, L) reduced from 67 ± 4 fT to 41 ± 3 fT (mean and standard deviation across experimental runs). The experimentally derived SNR of the gamma response decreased from 5.8 ± 1.0 to 4.2 ± 0.6

**Fig. 5.**
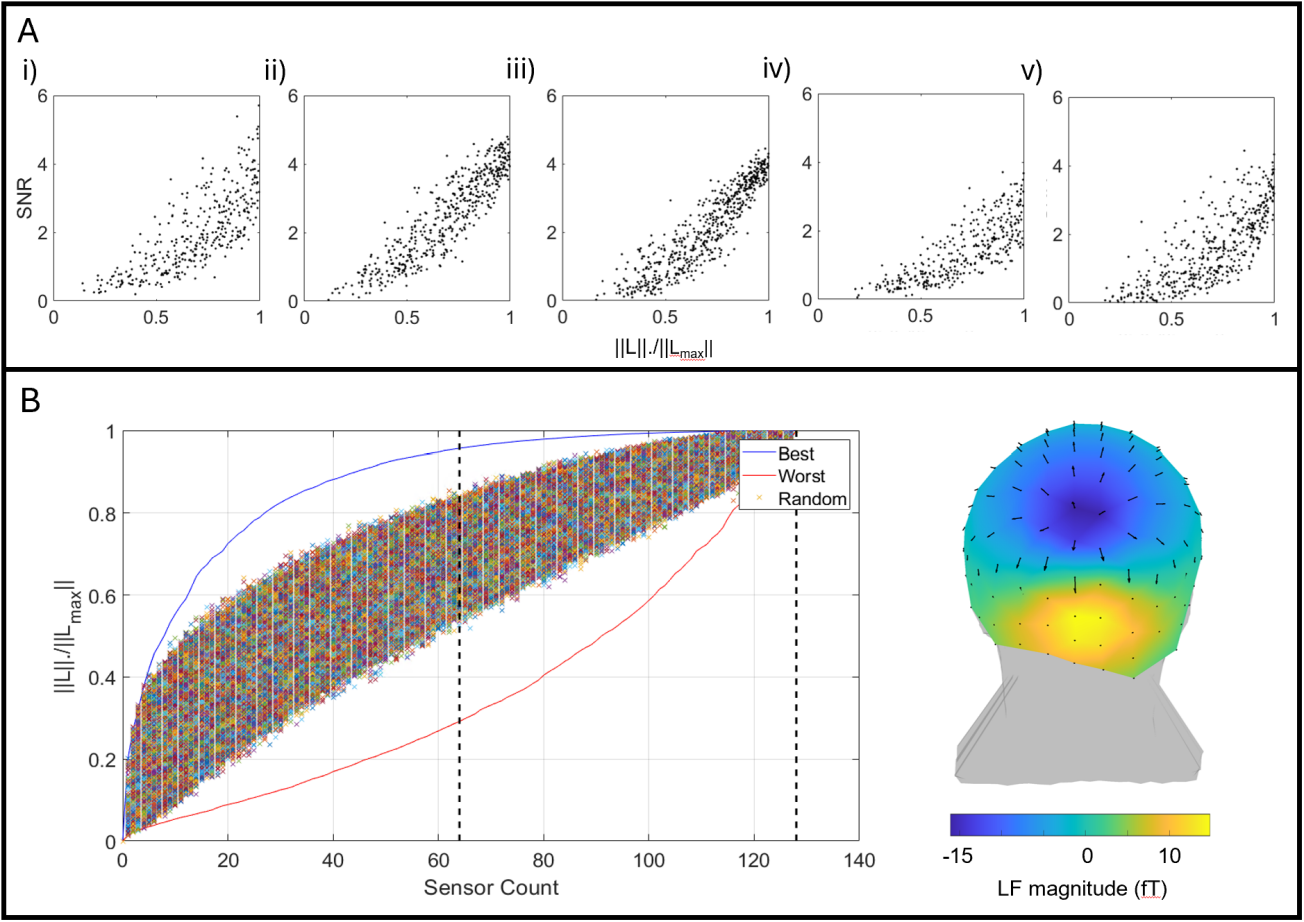
Channel count increases SNR. (A) SNR as a function of the total measurable signal (which is quantified as $\frac{\left\|\;\text{l}\;\right\|}{\left\|\;{\text{l}}_{\text{max}}\right\|}$ and varied randomly based on different sensor counts). All five experimental repeats shown separately; note the monotonic relationship. (B) Total measurable signal for a dipole in visual cortex as a function of channel count. The field pattern is shown in inset (the arrows show the field itself at each OPM, whilst the colours show the magnitude of the radial component with respect to the scalp). In the graph, the crosses show the case where different numbers of sensors are switched on/off randomly. The blue line shows the case where sensors switched on are located judiciously over visual cortex. The red line shows the case for poorly positioned sensors.

Figure 5A further quantifies the advantages of a high channel count by plotting SNR as a function of the total measurable signal. As noted in our Methods section, the total measurable signal is measured as the Frobenius norm of the forward model from the location of interest in visual cortex, ‖ l ‖. Here this quantity is divided by $\left\|\;{\text{l}}_{\text{max}}\right\|$, which represents the Frobenius norm calculated assuming all 163 available sensors in the helmet slots had been filled. (I.e., a value of $\frac{\left\|\text{l}\right\|}{\left\|\;{\text{l}}_{\text{max}}\right\|}=0.8$ denotes that we are measuring 80% of the signal that we could expect from a fully populated helmet.) Data from all five experimental repeats are shown (panels i–v). As expected (Hill et al., 2024), SNR increases monotonically with $\frac{\left\|\text{l}\right\|}{\left\|{\text{l}}_{\text{max}}\right\|}$, meaning SNR is improved with channel count.

However, increasing channel count is not the only way to change ‖l‖ and SNR. Figure 5B shows $\frac{\left\|\text{l}\right\|}{\left\|\;{\text{l}}_{\text{max}}\right\|}$ plotted as a function of the number of sensors used; the plot is shown for the specific case of a current dipole in visual cortex (at the location of the largest gamma change—the field pattern generated by this dipole is shown in inset). The crosses show values of $\frac{\left\|\text{l}\right\|}{\left\|\;{\text{l}}_{\text{max}}\right\|}$ derived when sensors were selected at random and switched off; it is clear that $\frac{\left\|\text{l}\right\|}{\left\|\;{\text{l}}_{\text{max}}\right\|}$ increases (approximately) with the square root of channel count, as would be expected. However, the blue line shows the case where sensors are judiciously located to maximise the measurable signal (i.e., sensors are strategically placed over visual cortex). Here, $\frac{\left\|\text{l}\right\|}{\left\|\;{\text{l}}_{\text{max}}\right\|}$ (and SNR) increases much faster with sensor count, and we can get within 5% of the highest possible signal with just 59 sensors. For completeness, the red line shows the case where sensors are poorly located. This shows that to achieve high sensitivity using an OPM-MEG array, we do not necessarily require a high channel count, if we can optimally distribute sensors over brain regions of interest. This will be addressed further in our discussion.

### 3.4 Human experiments: Movie watching

**Fig. 6.**
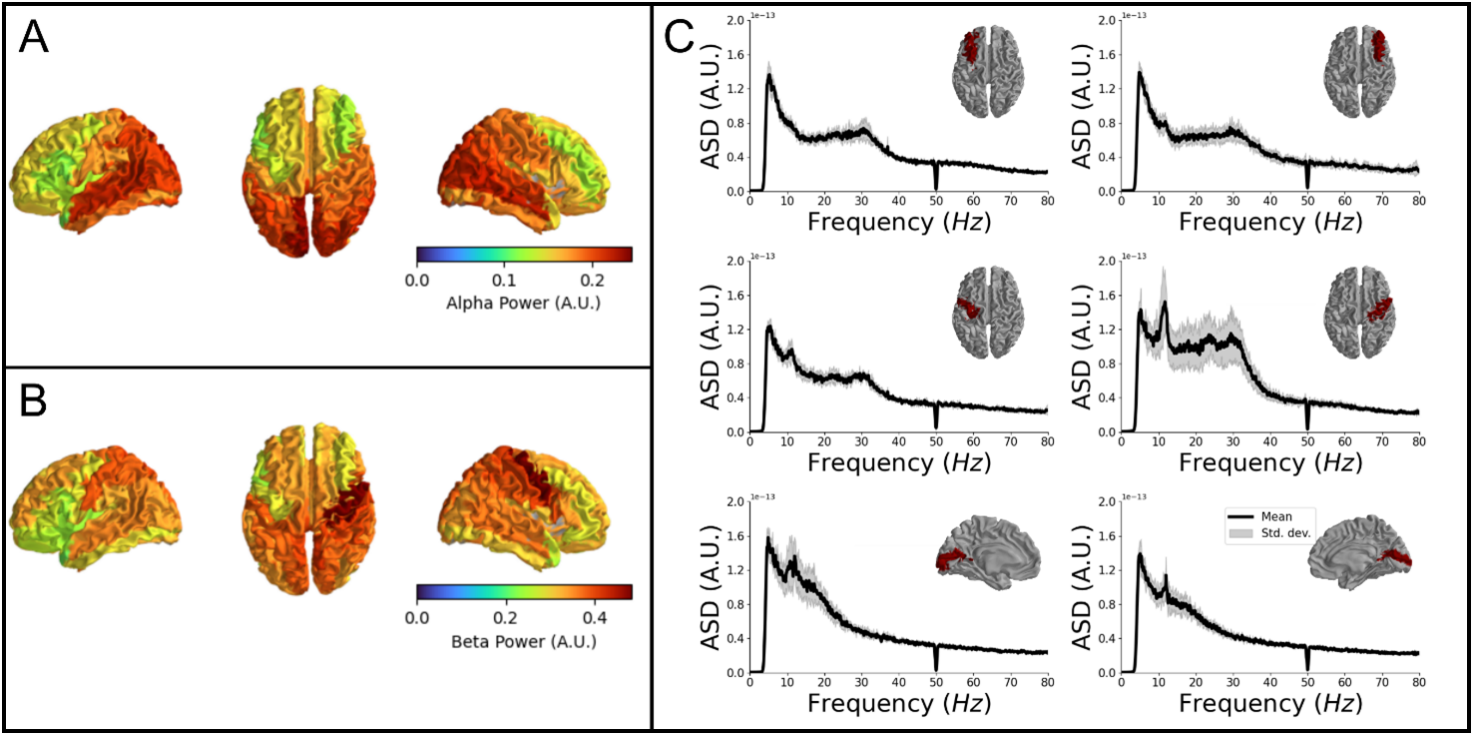
Resting-state oscillatory power. (A) Spatial distribution of relative alpha power plotted as a heat map for all AAL regions. (B) Spatial distribution of relative beta power. (C) Example power spectral density plots reconstructed for six AAL regions—left and right middle frontal gyrus, primary motor cortex, and primary visual cortex. Note the differences in power spectrum in different brain regions.

For the movie-watching data, we had 336 ± 16 working channels. We retained 615 ± 12 s of data free from artefacts. Figure 6A and B shows the spatial distribution of relative alpha band and beta band power across the brain, respectively. Both results have been averaged across experimental repeats. Relative alpha power was concentrated in the occipital and temporal lobes whilst beta power was maximal over the parietal lobes, in agreement with previous findings (Hunt et al., 2016). Figure 6C shows the amplitude spectral density for six regions in the brain; in all cases, the black line shows the mean PSD, whilst the grey shaded areas show the standard deviation across five repeats of the experiment. Occipital regions show a clear alpha peak in the spectrum, whilst sensorimotor areas have both prominent alpha and beta components. (Note that the large beta peak in sensorimotor regions reduces the relative contribution of alpha power to the overall spectrum—hence why the sensorimotor regions are not prominent in the relative alpha power map (Fig. 6A).

**Fig. 7.**
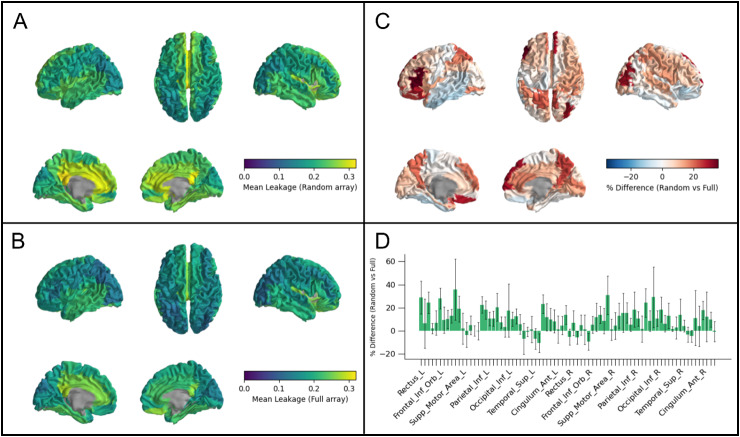
The advantages of high channel count for spatial resolution. (A) Signal leakage from all AAL regions for a randomly sampled array with ~192 channels. (B) Signal leakage for a full 336 ± 16-channel array. (C) The difference in signal leakage between the random and full arrays, plotted as a percentage (i.e., 100((random – dense)/dense)). Note that for most brain regions, the difference is positive meaning that the dense array is advantageous in terms of signal leakage. (D) Bar chart showing relative improvement (again as a percentage) in spatial resolution for all brain regions. Here, the bar height represents the mean for each region and the error bar shows standard deviation across five experimental runs.

Finally, Figure 7 shows the advantages of high channel count for spatial resolution. In panels A and B, the colours represent the magnitude of signal leakage from each region to all other regions. Larger values indicate more leakage, and, therefore, a lower spatial resolution. Leakage tends to be smaller for regions with good sensor coverage and worse for deeper regions as would be expected. However, the leakage is generally higher (lower spatial resolution) for the sparse array (Fig. 7A) than for the dense array (Fig. 7B). This is also shown in Figure 7C which delineates the percentage difference between leakage measured for the sparse and dense arrays. Note that the values in Figure 7C are positive for most brain regions, indicating that the addition of extra channels improves spatial resolution. This is further shown in the bar chart in Figure 7D which shows the relative improvement afforded by moving from the sparse to the dense array.

## 4 Discussion

OPM-MEG is providing a transformative shift in MEG instrumentation. However, its ultimate adoption to replace SQUID-based devices will depend on our ability to build high channel count systems (beyond the ~300 used for conventional MEG) that will maximise sensitivity, spatial resolution, and coverage. Such high-density arrays must offer characterisation of magnetic field distributions with a high degree of accuracy, whilst also maintaining the advantages—such as motion robustness and lifespan compliance—that have become important reasons for the success to date. In this paper, we have constructed a high channel count OPM-MEG device using triaxial sensors and a synchronised integrated miniaturised electronic control system. The platform is flexible, with the electronics able to expand (or contract) to support any arbitrary number of sensors. We have demonstrated a fast method for system calibration which ensures accuracy of measurements, and via phantom and human experiments, we have demonstrated viability of this platform.

The initial validation of our system was undertaken using a PCB-based phantom. This enables a means to gather data where the ground truths—in this case the distances between the five magnetic dipoles—are known with a high degree of accuracy (from the PCB manufacturing process). Localising dipoles represents an important test of any MEG system, since accurate localisation can only be achieved if the array offers high fidelity recording of a magnetic field distribution. We showed that, following calibration, we could determine the distances between dipoles on the phantom to an accuracy of ~1 mm. Furthermore, we showed that the correlation between the magnetic fields measured by the array, and those expected from the dipoles, agreed with a correlation coefficient >0.998.

This phantom result is important for three reasons: first, it *validates the sensors and the process used to calibrate them*. It is noteworthy that the calibration (via the matrix coil) and the validation method (using the phantom) are independent; whilst both work by imposing well-characterised fields on the array, those fields are very different. The fact that the relative errors in dipole positions are low and the correlations between the data recorded and the magnetic dipole model are high can only mean that the calibration is working. Indeed, data shown in Figure 3 show that without calibration, the agreement between the measured and modelled fields is degraded. Second, the high level of agreement suggests that our system is *not subject to systematic errors due to crosstalk*. Crosstalk affects both the gain and orientation sensitivity of a sensor, which in turn can affect localisation accuracy. It can be corrected by alternative designs of on-board sensor coil (Nardelli et al., 2019) but such designs typically add bulk to sensors and are limiting for practical high-density arrays. For open loop sensor operation, as long as the relative locations and orientations of the sensors remain constant during a recording, then the effect of crosstalk will also remain constant and is accounted for by our calibration. The high degree of accuracy demonstrated by our phantom experiment (with calibration) would be unlikely if our array was subject to crosstalk errors (crosstalk could be contributing to errors in the uncalibrated data). Finally, *our calibration process is both simple and practical* compared with previous studies (Hill et al., 2025). A limitation of calibration using an external coil (like our matrix coil approach) is that, if the array moves during calibration, then the accuracy of the result is degraded. However, here our calibration requires just 1-s of data (see Appendix A1). This should be possible to acquire even when scanning difficult participants (e.g., children) who find it hard to keep still, since data could be recorded for a longer duration alongside head tracking. The calibration itself could then be performed using the 1-s window with the smallest subject motion. Because this is possible whilst the helmet is being worn, it can be adapted for use with flexible OPM helmets where sensor locations change, subject-to-subject.

Our phantom has two limitations. First, we had no means to position the phantom in a known location relative to the sensor array. Consequently, our analysis was limited to measurement of relative errors in the position of the dipoles rather than absolute errors in their location (relative to the array). Whilst relative errors remain a useful measure of system performance, it is the absolute error that is of interest in most MEG studies. Developing phantoms where the absolute location of the dipole is known *a priori* should be a topic of future work. Second, here we chose to build a phantom based on magnetic dipoles, rather than current dipoles. (The former arises from a circulating current (i.e., a loop of wire) whilst the latter consists of a current that flows between two points separated by a short distance.) A current dipole is a closer approximation to fields from the brain and is, therefore, typically used in MEG. However, current dipoles are also hard to make, and practical examples usually employ a current flowing along a finite distance (usually ~5–10 mm), whereas the equivalent current dipole model assumes the current exists at a single point in space. This introduces an intrinsic uncertainty in dipole location compared with the ground truth. In contrast, a magnetic dipole is easy to make (using a spiral current path) and the equivalent magnetic dipole model should localise to a single point at the centre of the spiral, which is easy to pinpoint with high accuracy. It is for this reason that we chose a magnetic dipole model. This generated known fields against which the accuracy of our sensor array could be benchmarked. However, current dipoles remain a closer match to “brain-like” fields and a phantom exploiting this architecture would be desirable for future OPM-MEG benchmarking.

Our visual experiment demonstrated the utility of a high-density device to detect gamma oscillations. Results showed that stimulus-induced modulation of gamma activity localised to the primary visual areas. Further, TFS data showed a gamma response at 40–50 Hz with an additional response at ~30 Hz. These were visible during presentation of the static grating and became stronger during the drifting grating. This is in agreement with the literature (Fries et al., 2008; Hill et al., 2024; Muthukumaraswamy et al., 2010). Generally, these effects can be seen anywhere in the 30 Hz to 100 Hz frequency range, although their precise spectral distribution differs between subjects and varies systematically with other factors (e.g., age; Rhodes et al., 2025). For this reason, the precise pattern observed here (e.g., separate 30 Hz and 40–50 Hz responses with an apparent cut-off at 60 Hz) is likely to be specific to the individual subject (rather than the instrument used).

Importantly, the gamma experiment showed the advantages of high-density arrays. Our previous work (Hill et al., 2024) used theory, simulation, and experiments to demonstrate a monotonic relationship between the total signal measurable by an array (‖l‖) and the SNR of the resulting beamformer reconstructed signal. Here, our results followed the same relationship, with gamma SNR again improving with ‖l‖ which was increased by adding channels up to a maximum of 349. Between a 165-channel (mean) array and 349-channel (mean) array, ‖l‖ increased by a factor of ~1.6; percentage change in gamma response increased by a factor of ~1.5, and SNR increased by a factor of ~1.4. However, we stress that increasing the total channel count is not the only way to increase ‖l‖. In cases where the brain region of interest is known a priori, it is possible to achieve the same sensitivity benefits by simply redistributing a smaller number of sensors over a smaller area of the scalp. For example, data shown in Figure 5 suggest that via judicious sensor placement, one can reach 95% of the SNR of a 384-channel array using just 177 channels. This is one of the significant advantages of OPMs over SQUIDs and shows that high channel count is not always required (e.g., in cases where a particular task is known to activate a specific region, or potentially in some clinical scenarios such as epilepsy, where seizure semiology may provide an a priori region for further investigation). However, in cases where whole brain coverage is required, then obviously high channel counts is necessary.

The movie-watching data also demonstrate the utility of our high-density system, with results in Figure 6 showing the expected distribution of alpha and beta power across the occipital and parietal lobes, respectively (e.g., see Hunt et al. (2016) for a comparison with conventional MEG). Moreover, the movie-watching results show the utility of a higher channel count to improve spatial resolution. We chose to characterise spatial resolution in terms of the leakage between regions across the AAL parcellation. Such leakage is a function of (1) the correlation between forward models from two regions and (2) the SNR of the reconstruction for each region. Forward model correlation is not impacted by sensor count (though it is reduced by moving sensors closer to the scalp—giving OPMs an inherent advantage over SQUIDs; Boto et al., 2019). However, SNR (as evidenced by our gamma band experiments) is impacted by high sensor density and is also enhanced by accurate calibration (Hill et al., 2025). It is for this reason that we likely see decreased signal leakage with higher number of channels. Importantly, this increase (in spatial resolution) was not uniform for all brain regions. For example, most notably, the temporal lobes showed little improvement with increased channel density. This is likely down to coverage since, even in our dense array, the temporal lobes were covered relatively poorly. In general, however, the effect of high-density sensors was positive and this will be important in future studies, for example, when measuring whole brain network connectivity.

The helmet housing the sensors was a 3D-printed structure bespoke to a single individual (made based on their MRI scan). It ensured that sensors were positioned as close as possible to the scalp. However, it has limitations which should be acknowledged. First, the sensors are heated, consequently the helmet becomes warm during operation and the design did not provide active cooling or insulation. To prevent discomfort to the participant, the array was rebooted between experiments and allowed to cool down. This was sufficient for our proof-of-principle demonstration. However, if high-density arrays are to be used for long periods (e.g., when trying to detect epileptic spikes in patients), then helmets will need to incorporate improved insulation or active cooling (e.g., by passing water (Pang et al., 2022) across the sensors to remove heat). In addition, the bespoke helmet is limited to a single participant and is impractical for large group studies, where generic (flexible) helmets that adapt to different head shapes are preferred. This raises the question of “coregistration” (i.e., how to determine where the brain is relative to the sensor array). In this paper, we used calibration to determine OPM locations relative to each other and then matched this to the CAD file for the bespoke helmet; because the helmet was based on an MRI, this automatically provided the location of the brain in the same coordinate frame as the sensors. In a generic helmet, however, a different coregistration procedure would be required; several are available—for example, using electromagnetic coils placed at anatomical landmarks, whose locations can be identified on MRI and localised using the sensor array. Alternatively, one can gather the location of optical markers on the array, alongside the shape of the face, using 3D optical scanning and then match the face shape to the MRI scan to determine the location of the brain. A different option might even use the calibration procedure, with a small number of OPMs placed on anatomical landmarks that can be identified by calibration, and their location matched to MRI. In sum, an extension to generic helmets for high-density arrays and better insulation/active cooling, and accurate coregistration should all form the topics of future work.

An additional concern of high-density arrays is that the combined weight of the sensors becomes large and makes wearable arrays impractical—particularly for use in children who may find it difficult to support the weight on their heads. However, the total weight of the 128 sensors was 512 g, and the total helmet weight (when populated with sensors) was 974 g. This is only marginally heavier than helmets used in previous studies in both children and adults (Rhodes et al., 2025; Rier et al., 2024) where weight ranged from 850 to 900 g. It is, therefore, likely that the weight of the present helmet would not present a limitation, but if this were the case, tethering the helmet to the ceiling of the MSR via an elasticated cord potentially offers a means to help participants (particularly children) support the weight. Similarly, the form factor of the electronics in the present study was not optimised and simply comprised two separate electronics boards which were independently powered and synchronised by a separate signal generator. The electronics boards were each 0.36 × 0.20 × 0.06 m and weighed 0.81 kg each. The two boards could, therefore, easily be integrated into a single package which could be mounted within the patient support or even worn by the participant as a backpack to enable ambulatory studies.

Finally, in the present study, we had a relatively large number of channels that were eliminated from our final analyses. This was caused by two separate issues: first, a few sensors were among the first triaxial OPMs built. They were handmade (rather than manufactured) and consequently they are less robust than newer sensors. Second, some sensors used a ribbon cable which attaches via a small clamp on the top of the sensor; as sensors age, this can become loose and consequently sensors become detached during a scan. Both of these issues are solved with more recent sensor designs (the latter problem being solved by a cylindrical (rather than ribbon) cable and a push-fit connector (rather than a clamp)).

## 5 Conclusion

We have successfully constructed an OPM-MEG array with >300 channels, using triaxial sensors and integrated miniaturised electronic control units. We have developed and applied new calibration methods to ensure that the fields measured are high fidelity and we have introduced a phantom that provides quantification of that fidelity. We have demonstrated our system by measuring MEG data during a visual task and a movie-watching paradigm, and we have demonstrated the advantages of high channel count for sensitivity and spatial resolution. The introduction of the first OPM-MEG system with a channel count higher than that of SQUID-based devices is a significant step on the path towards OPMs becoming the sensor of choice for MEG measurement.

## Figures and Tables

**Appendix Fig. A1.**
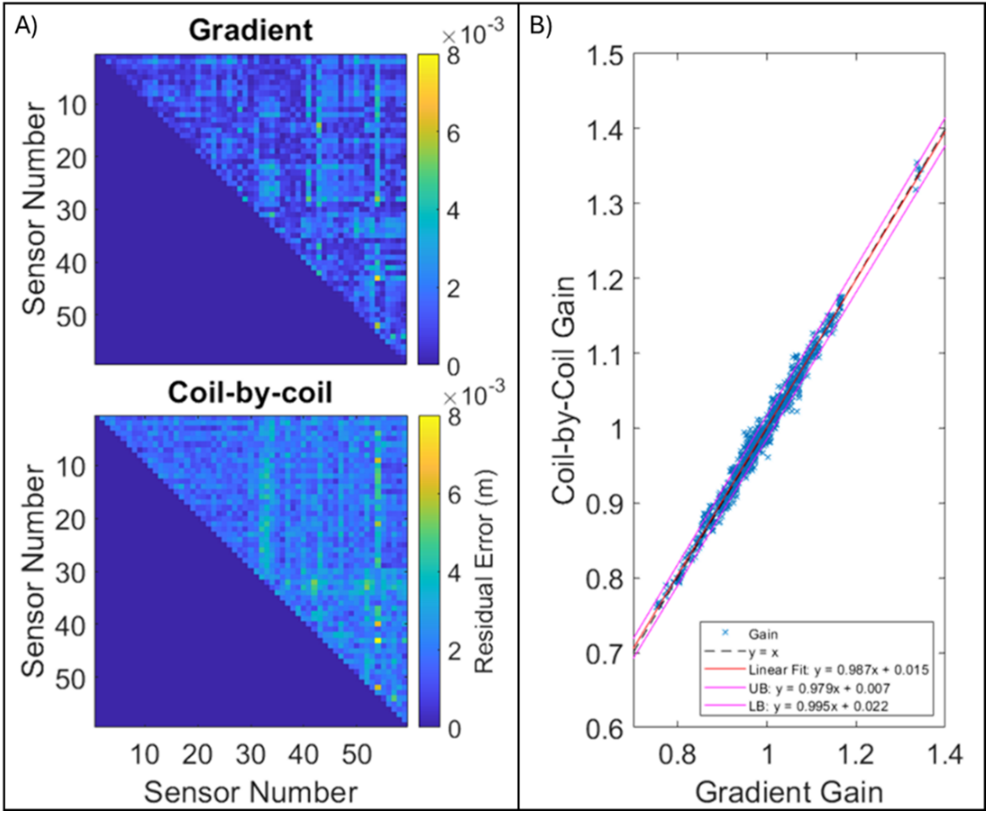
Comparison of calibration techniques: (A) Matrices showing the mean (across experimental repeats) sensor-to-sensor distance error between CAD and calibration. The gradient method is shown in the upper panel and coil-by-coil method is shown in the lower panel. (B) Channel gains determined by the gradient and coil-by-coil calibrations, plotted against each other (all channels and all runs overlaid). The black dashed line shows the line of equality; the red line shows a linear fit. The close relationship suggests that the two calibration methods result in similar gain measures.

**Appendix Fig. A2.**
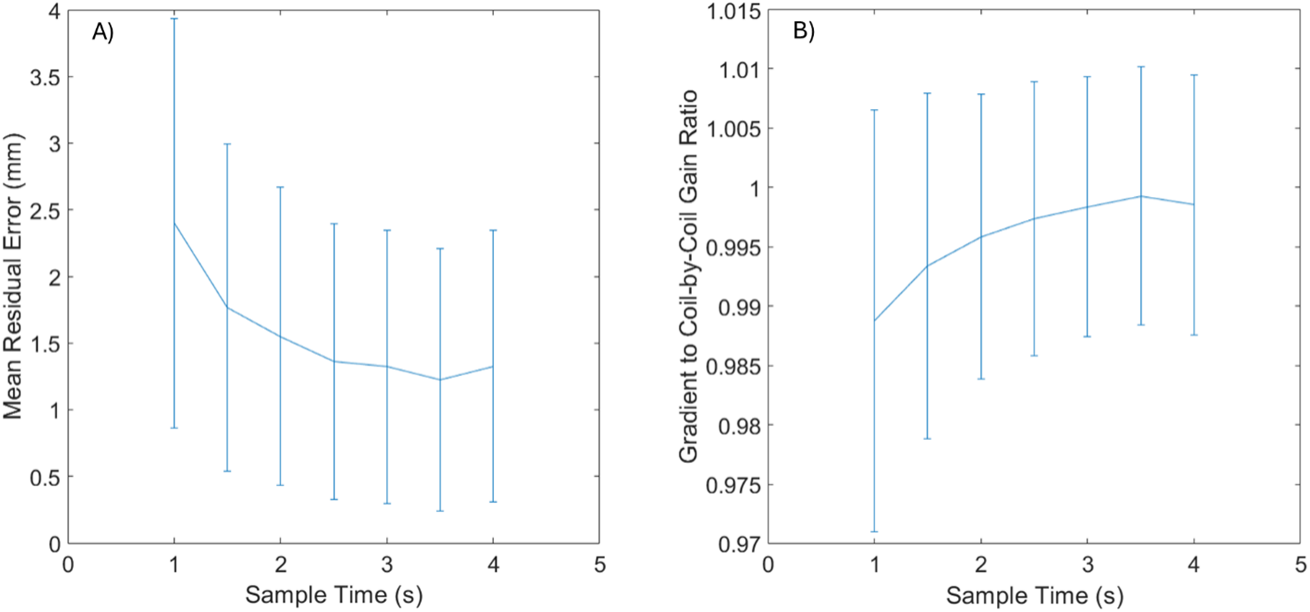
Accuracy as a function of sample time. (A) The average error between gradient calibration and CAD, plotted as a function of the amount of data used (sample time) for the calibration. (B) The average ratio of the gradient and coil-by-coil-derived gains, plotted as a function of sample time for gradient-based calibration. In both (A) and (B), the error bars show the standard deviation across runs.

## Data Availability

All data used to produce the results presented here is available via Zenodo 10.5281/zenodo.17807938. The software used for data analysis is available at https://github.com/HollySchofield/Towards-a-384-channel-magnetoencephalography-system-based-on-optically-pumped-magnetometers.git.

## Appendix A1: Further Coil Calibration Results

The paper describes a calibration technique in which matrix coils are used to generate uniform fields and linear gradients, and a fitting algorithm is used to determine the sensor positions, orientations, and gains. To further investigate performance of this method, we undertook two additional experiments. First, we aimed to compare our “gradient fitting” calibration with a more established “coil-by-coil” method (originally described in Hill et al., 2025). Second, we aimed to determine how the amount of data used for the gradient fitting approach affected the overall accuracy of the calibration.

### A1.1. Method

#### A1.1.1. Calibration comparison

The coil-by-coil method has been described in detail in a previous paper. Briefly, a forward model describing the field per unit current generated by each matrix coil element was created, within a volume of interest across the centre of the MSR (this was done once, using a flux gate magnetometer to accurately characterise the fields). To perform a calibration, we generated sinusoidally modulated fields, simultaneously using 96 coil elements, with each element generating a different frequency (between 2 and 11.5 Hz with a separation of 0.1 Hz). We captured 20 s of data from all OPM channels in the array. A Fourier approach was used to isolate signals from each coil element, giving the contribution of each coil to data recorded at each OPM-MEG channel. By fitting these data to the forward models for the coils, it becomes possible to determine the location, orientation, and gain of the channel. This can then be repeated for all channels.

To compare the coil-by-coil and gradient approaches, we mounted 64 triaxial sensors in a 3D-printed OPM-MEG helmet (Cerca Magnetics Ltd., Nottingham, UK). The helmet was placed close to the centre of the MSR and we ran both calibration procedures six times. We extracted sensor positions using each technique and computed a matrix of the distances between each possible sensor pair in the array. We then subtracted these matrices from the expected values from the CAD model of the helmet to produce residual matrices showing the “error” in calibration-derived distances (i.e., taking the CAD as the ground truth). In addition, we took the calibration-derived gain values from the coil-by-coil method and plotted them against equivalent values from the gradient approach.

#### A1.1.2. Quantity of data

For the coil-by-coil method to work, we required a relatively long data segment (20 s in this case), to get the spectral resolution required in Fourier space to separate signals from 96 coil elements. However, using the gradient method, we require just eight signals (three uniform fields and five gradients). Consequently, the data segment required can be much shorter. To investigate how the amount of data (i.e., the speed at which the gradient method can be run) affects the accuracy of the calibration, we took the data from the experiment described above (which used a 4-s data segment) and sequentially reduced the amount of data used for the calibration process (to 1 s in steps of 0.5 s).

### A1.2. Results

Appendix Figure A1A presents the error matrices; the difference between the gradient method and CAD is shown in the upper panel; the difference between the coil-by-coil method and CAD is shown in the lower panel. In both cases, data are averaged across all six runs of the experiment. The average residuals across all sensor pairs were 1.3 ± 0.9 mm and 2.0 ± 0.8 mm (mean ± standard deviation across all matrix elements) for the gradient and coil-by-coil calibrations, respectively. The average residual between the two calibrations was 2.1 ± 0.7 mm. These results indicate good agreement between the calibration methods and the ground truth (CAD) and good agreement with each other.

Appendix Figure A1B shows the channel gains derived from two calibrations plotted against each other (data for all experimental runs are shown on the same plot). There is a clear linear relationship which sits close to the line of equality (shown in black). Quantitatively the gradient of the line was 0.987 and the intercept was 0.015, which is close to a perfect agreement (i.e., values of 1 and 0 for gradient and intercept, respectively).

In Appendix Figure A2, panel A shows the mean (across sensors) distance error in gradient calibration-derived sensor positions (compared with CAD), as a function of the total amount of data used for calibration. The error bar shows standard deviation across sensors. Even for 1 s of data, the distance error is only 2.5 mm which is smaller than the minimum accuracy of <4 mm, suggested by Zetter et al. (2018) as the requirement to be comparable with cryogenic MEG systems.

Panel B shows the ratio between the gain values derived using gradient and coil-by-coil calibration methods (i.e., a value of 1 would indicate perfect agreement between the two techniques). The figure shows the mean ratio (across channels) plotted as a function of the amount of data used for the gradient calibration method; the error bar shows standard deviation across channels. Again, even with 1 s of data, the discrepancy between gains is less than 1.5%.

### A1.3. Discussion

This additional experiment shows agreement between our “gradient” calibration method and a more established technique. Moreover, it shows that accurate calibration can be achieved using just 1 s of data, meaning that gradient-based calibration can be employed in realistic situations. One disadvantage of matrix-coil based calibrations is that if the head moves during calibration, the sensors see a changing field and the accuracy of the sensor locations, orientations, and gains is diminished. Expecting participants (especially children) to sit still for a 20-s data collection is unreasonable. However, with accurate results achievable in just 1 s, the technique becomes much more practical. For example, even in participants who find it hard to keep still, the calibration process could be run at multiple intervals and, if paired with head tracking, experimenters could isolate the 1-s window with the lowest head movement. Multiple rapid calibrations could also be used to assess sensor stability at regular intervals. In sum, we believe this rapid and simple calibration method is a useful addition to OPM-MEG systems.
